# PDZ2-conjugated-PLGA nanoparticles are tiny heroes in the battle against SARS-CoV-2

**DOI:** 10.1038/s41598-024-63239-w

**Published:** 2024-06-06

**Authors:** Noah Giacon, Ettore Lo Cascio, Valeria Pennacchietti, Flavio De Maio, Giulia Santarelli, Diego Sibilia, Federica Tiberio, Maurizio Sanguinetti, Wanda Lattanzi, Angelo Toto, Alessandro Arcovito

**Affiliations:** 1 Dipartimento di Scienze Biotecnologiche di Base, Cliniche Intensivologiche e Perioperatorie, Università Cattolica del Sacro Cuore, Largo F. Vito 1, 00168 Rome, Italy; 2 Dipartimento di Scienze Biochimiche “A. Rossi Fanelli”, Laboratory Affiliated to Istituto Pasteur Italia - Fondazione Cenci Bolognetti, Sapienza Università di Roma, P.le A. Moro 5, 00185 Rome, Italy; 3 Dipartimento di Scienze di Laboratorio e Infettivologiche, Fondazione Policlinico Universitario “A. Gemelli”, IRCCS, Largo A. Gemelli 8, 00168 Rome, Italy; 4 Dipartimento di Scienze della Vita e Sanità Pubblica, Università Cattolica del Sacro Cuore, Largo F. Vito 1, 00168 Rome, Italy; 5 Fondazione Policlinico Universitario “A. Gemelli”, IRCCS, Largo A. Gemelli 8, 00168 Rome, Italy

**Keywords:** Functionalized PLGA-based nanoparticles, SARS-CoV-2 envelope protein, Virus–host interaction, Human PDZ2-ZO1, Biochemistry, Microbiology, Virology, Nanobiotechnology

## Abstract

The COVID-19 pandemic caused by SARS-CoV-2 has highlighted the urgent need for innovative antiviral strategies to fight viral infections. Although a substantial part of the overall effort has been directed at the Spike protein to create an effective global vaccination strategy, other proteins have also been examined and identified as possible therapeutic targets. Among them, although initially underestimated, there is the SARS-CoV-2 E-protein, which turned out to be a key factor in viral pathogenesis due to its role in virus budding, assembly and spreading. The C-terminus of E-protein contains a PDZ-binding motif (PBM) that plays a key role in SARS-CoV-2 virulence as it is recognized and bound by the PDZ2 domain of the human tight junction protein ZO-1. The binding between the PDZ2 domain of ZO-1 and the C-terminal portion of SARS-CoV-2 E-protein has been extensively characterized. Our results prompted us to develop a possible adjuvant therapeutic strategy aimed at slowing down or inhibiting virus-mediated pathogenesis. Such innovation consists in the design and synthesis of externally PDZ2-ZO1 functionalized PLGA-based nanoparticles to be used as intracellular decoy. Contrary to conventional strategies, this innovative approach aims to capitalize on the E protein-PDZ2 interaction to prevent virus assembly and replication. In fact, the conjugation of the PDZ2 domain to polymeric nanoparticles increases the affinity toward the E protein effectively creating a “molecular sponge” able to sequester E proteins within the intracellular environment of infected cells. Our in vitro studies on selected cellular models, show that these nanodevices significantly reduce SARS-CoV-2-mediated virulence, emphasizing the importance of exploiting viral-host interactions for therapeutic benefit.

## Introduction

**Figure 1:**
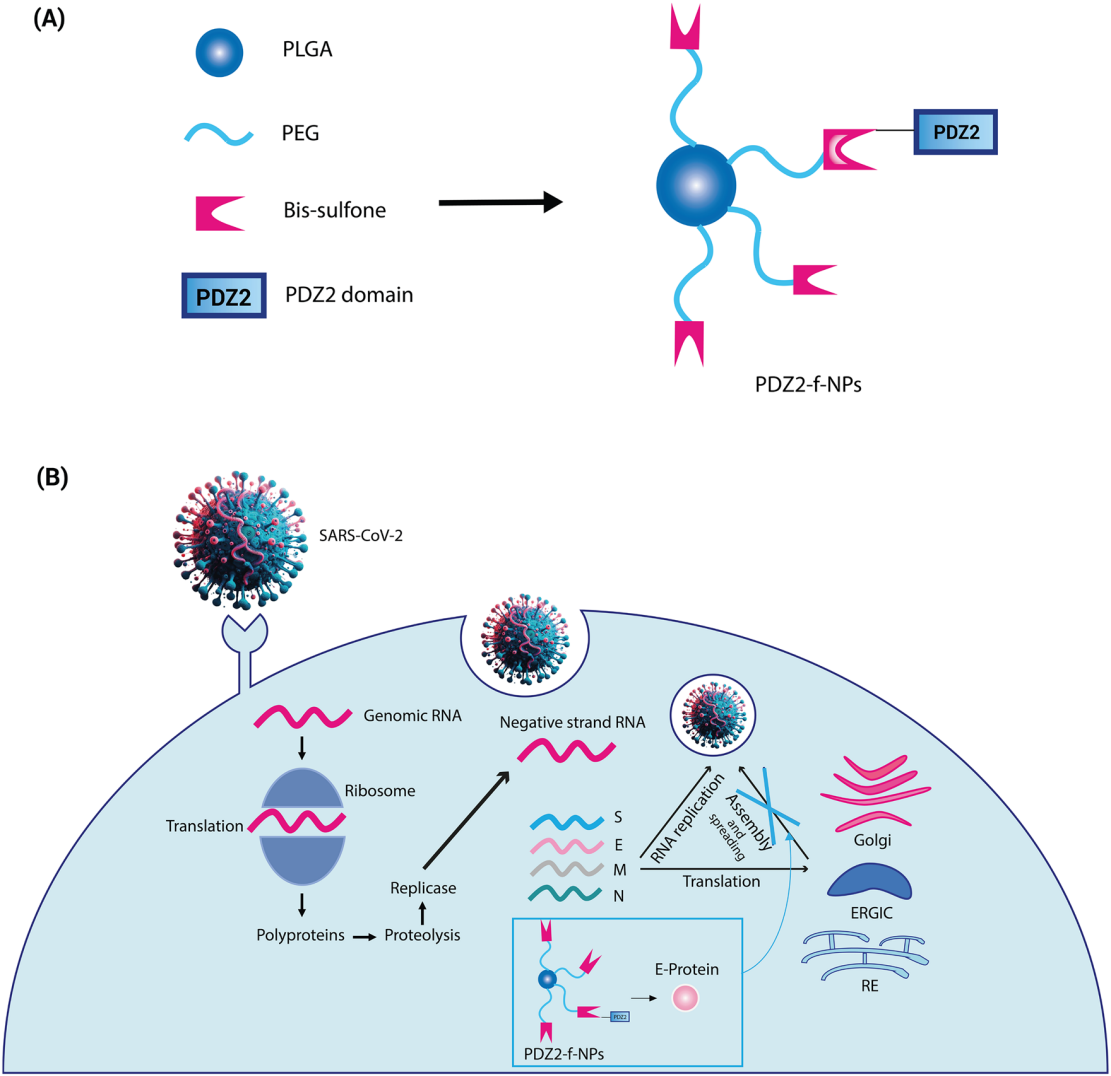
(**A**) Schematic illustration of PDZ2-f-NPs. (**B**) Scheme of virus infection and nanoparticle-mediated interference with this mechanism.

For years, Coronaviruses were considered simple seasonal respiratory viruses, but they have proven responsible for diseases with significant human morbidity and mortality. The emergence of the new disease caused by SARS-CoV-2, named “Coronavirus Disease 2019” (Covid-19), swiftly escalated to a global scale, leading the World Health Organization (WHO) to declare a pandemic status on March 11, 2020^1^. SARS-CoV-2 is an enveloped single-stranded positive RNA virus. This virion is characterized by the expression of four key structural proteins: Spike protein (S), Membrane protein (M), Envelope protein (E), and Nucleocapsid protein (N)^2^. The E protein is the most enigmatic and smallest among the various structural proteins that constitute Coronaviruses and plays a multifunctional role in virus pathogenesis, assembly, and release (see Fig. 1). SARS-CoV-2 E-Protein is a small membrane polypeptide that can exist in either monomeric or homo-pentameric form and together with the M and S proteins it forms the viral envelope^3–5^. One of its peculiarities is to act as a viroporin facilitating the production, maturation, and release of the virus from infected cells^3^. In addition, when E protein is in its monomeric form, it can influence intracellular activities through its C-terminal domain, whose β-coil-β structure appears to determine its localization in the Endoplasmic Reticulum (RE), Golgi, and endoplasmic-reticulum-Golgi intermediate compartment (ERGIC), where it participates in virus assembly and budding^6^.

SARS-CoV-2 E-protein possesses, at its C-terminus, a PDZ-binding motif (PBM). Such a motif, consisting of 4 amino acid residues (DLLV) can bind different PDZ domain containing proteins, such as PALS1 (Protein Associated with Caenorhabditis Elegans Lin-7 protein 1)^3,5–12^, and ZO-1 (Zonula Occludens-1)^13,14^, both involved in the formation and maintenance of epithelial cell tight junctions as well as for cell communication, adhesion, and the regulation of barrier functions in tissues^15–23^. Importantly, decreased virulence could be achieved by selectively eliminating the PBM from the E protein, underscoring the crucial involvement of PDZ-dependent viral targeting of host proteins^10^. The significance of the PBM in the E protein was evidenced in cells infected with SARS-CoV, where the removal of the PBM led to the development of an alternative PBM following multiple cell passages^24^.

PDZ domains represent one of the largest family of protein–protein interaction domains in the human proteome and are named after the first three proteins in which they were discovered, PSD-95, Dlg1 and ZO-1. These domains are involved in the assembly of protein complexes by binding to short linear peptide motifs present in the target proteins, usually located at the C-terminus of ligands^25^. The PDZ2 domain of ZO-1 (PDZ2-ZO1) possesses a pivotal role in mediating the interactions with various binding partners, contributing to the assembly and stabilization of tight junctions and gap junctions^26–29^. Moreover, PDZ2 domain is capable of binding to the E protein of different viruses, including SARS-CoV-2^13,26^. In a prior investigation, the affinity between the PDZ2-ZO1 and the PBM of SARS-CoV-2 was assessed, yielding a K_D_ value of 2 μM^13^.

Given the notable affinity between the PDZ2-ZO1 domain and the E protein, we devise an innovative therapeutic method utilizing PDZ2 functionalized nanoparticles (PDZ2-f-NPs) to harness this interaction to mitigate virus-mediated infection. By capitalizing on the interaction between the C-terminal region of E protein and the PDZ2 domain, these functionalized nanoparticles have the potential to impede virus assembly, thereby slowing down the progression of infection (see Fig. 1). The necessity of conjugating these protein domains to a cargo is mandatory as the selected PDZ2 domains would not cross the plasma membrane^30,31^; moreover, larger constructs made of different polymers have significantly higher circulation time, thus favoring a sustained uptake in the target cells^32^. Therefore, to reach this goal, PDZ2-f-NPs have been developed, featuring a polylactic-co-glycolic acid (PLGA) core and outer polyethylene glycol (PEG) arms. PLGA is a polyester widely used in medicine, indeed it is biodegradable, bioavailable, and FDA-approved for use^33–37^; while PEG is a hydrophilic polyether known for its ability to increase the circulating lifetime of nanoparticles and reduce their immunogenicity^38–40^. NPs conjugation was made exploiting PEG arms which have been linked to bis-sulfone, a benzene acid derivative with two sulfonic functionalization, that is capable under specific conditions of generating Michael’s reagents^41^.

Developed by Brocchini’s group, bis-sulfone presents a selective and efficient approach for PEGylation of disulfide bonds in proteins. This method can be extended to C- or N-terminal histidine tags (His-tags), commonly used in protein purification and expression. The conjugation of histidine with bis-sulfone occurs through an addition–elimination mechanism, akin to thiol conjugation, resulting in a bridged conjugate^41–43^.

**Figure 2:**
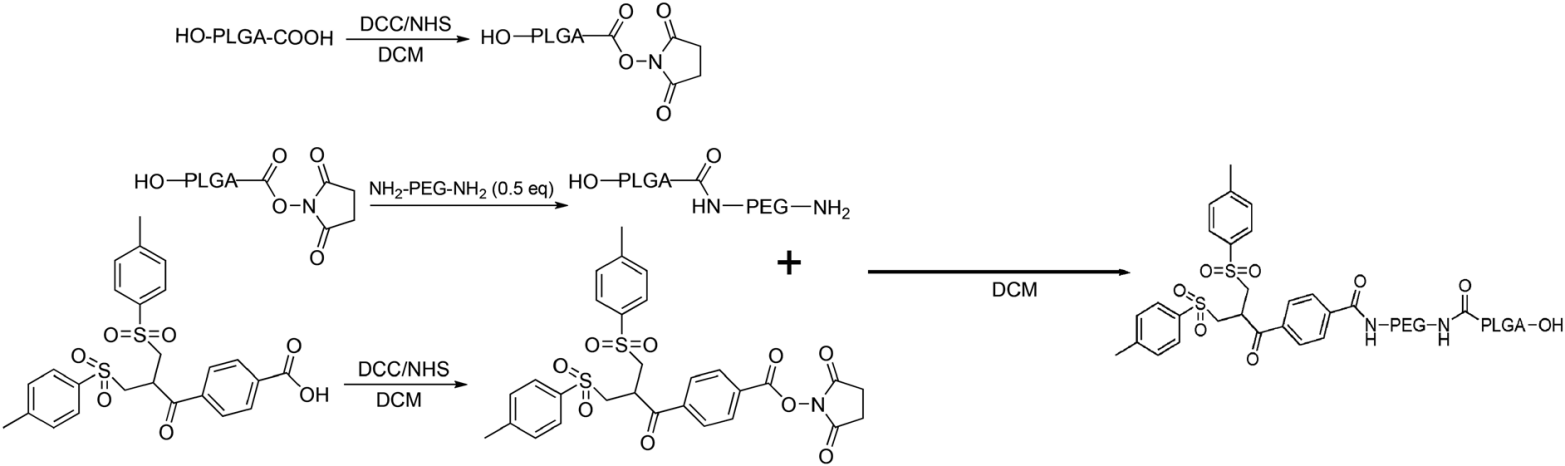
Schematic Illustration of PLGA-PEG-Bis-sulfone synthesis.

The resulting PLGA-PEG-bis-sulfone nanoparticles act as a platform for attaching specific targeting moieties and molecules onto their surface. Once the polymer was synthesized (see Fig. 2), nanoparticles (NPs) were obtained via nanoprecipitation technique^44–46^ and were induced to release toluene-sulphonic acid at basic pH; in this way a α, β-unsaturated carbonyl can be obtained. This reactive moiety can then undergo a Michael addition reaction with both thiol and imidazole groups^41–43^. In this context, the *his-tagged PDZ2* domain needs to be chemically linked to the bis-sulfone moiety.

To monitor cell uptake, we synthetize 6-Coumarin-loaded-PDZ2-f-NPs to visualize them via confocal microscopy (see Fig. 6). Thanks to this study we had been able to see that our NPs can be internalized in VERO cells; hence, we studied in vitro how SARS-CoV-2 infection spreads in the presence of PDZ2-f-NPs (see Fig. 7). This study delves into the potential of the E protein-PDZ2 domain interaction for a novel antiviral strategy, paving the way for new avenues in antiviral research and offering valuable insights into manipulating viral-host interactions for therapeutic benefit.

## Methods

### Synthesis of PLGA-PEG co-polymer

The synthesis of PLGA-PEG copolymer followed the method outlined by Zumaya et al., with minor modifications^47^.

### PLGA-NHS activation

PLGA-COOH (1 g, MW 10kDa) (Nanosoft polymers, Winston-Salem, NC, USA) was activated and converted to PLGA-NHS with excess of *N,N*′-Dicyclo-hexylcarbodiimide (DCC) and *N*-Hydroxysuccinimide (NHS). Quickly, PLGA-COOH was dissolved in DCM (10 ml, obtaining a solution of 0.01 mM) (Merck, Darmstadt, DE) followed by the addiction of 2.5 equivalent of DCC (52 mg, 0.025 mM) and NHS (29 mg, 0.025 mM) (both from Fluorochem Ltd, Glossop, UK). The reaction was left under magnetic stirring at room temperature overnight. Insoluble dicyclohexyl urea was filtered and the activated PLGA was dried under vacuum at room temperature to be later conjugated to PEG (see Fig. 2).

### PLGA-NHS conjugation through NH_2_-PEG-NH_2_

The resultant PLGA-NHS was dissolved in 10 ml of DCM before the addiction of 2 equivalent of NH2-PEG-NH2 (MW 3 kDa) (Sigma-Aldrich, Saint Louis, MO, USA) obtaining a solution of 0.02 mM. Again, the reaction was left under magnetic stirring overnight and the resultant polymer was dried under vacuum. To separate NH2-PEG-NH2 from PLGA-PEG copolymer, the obtained dried product was purified through solid–liquid extraction with methanol (Fluka Chemicals, Buchs, CH) in order to get ready for the next reaction (see Fig. 2). Subsequently the compound was characterized by Fourier-transform infrared spectroscopy (FT-IR) (see Fig. S1).

### Bis-sulfone activation

100 mg of Bis-sulfone (Fluorochem Ltd, Glossop, UK) were dissolved in 10 ml of DCM (0.02 mM). Both DCC and NHS were added in a stoichiometric excess of 2 times (0.04 mM) compared to Bis-sulfone. The reaction was left under gentle stirring for about 2 h and the resulting product was filtered, dried under vacuum, and lastly purified via solid–liquid extraction using diethyl ether (Merck, Darmstadt, DE). This process effectively removed unreacted Bis-sulfone, separating it from the desired product (see Fig. 2).

### Preparation of PLGA-PEG-Bis-sulfone

After the activation of bis-sulfone, PLGA-PEG-NH2 were dissolved in 10 ml of DCM (0.007 mM). Once solubilized, 1.1 equivalents of activated bis-sulfone were added to the solution (0.0077 mM). The reaction was allowed to proceed under magnetic stirring over-night. The resulting polymer was purified via solid–liquid extraction with methanol and dried under vacuum to be later finally used to prepare NPs (see Fig. 2). The purity of compound was characterized by FT-IR and Nuclear Magnetic Resonance (NMR) (see Figs. S1, S2).

### Preparation of PLGA-PEG-Bis-sulfone nanoparticles

To synthetize PLGA-PEG-Bis-sulfone nanoparticles (PPB-NPs), we performed nanoprecipitation method. PLGA-PEG-Bis-sulfone was dissolved in Tetrahydrofuran (THF) (Applied Biosystems by Thermo Fisher Scientific, Waltham, MA, USA) to reach a concentration of 100 mg/ml. Finally, 300 μl (equivalent to 30 mg of PLGA-PEG-bis-sulfone polymer) of the result solution was added dropwise to 7 ml of stirring water. The final product was freeze-dried and then analyzed through DLS and NTA.

### Preparation of PEG-mono-sulfone

The synthesis of PEG-mono-sulfone was induced adding to freeze-dried-NPs an appropriate buffer (1500 mM of NaCl, 20 mM EDTA (Sigma-Aldrich, Saint Louis, MO, USA) and 500 mM of sodium phosphate (Carlo Erba, Val de Reuil, FR), pH 8)^41^. The reaction was incubated at 37 °C for ~ 6h.

### Protein expression and purification

Expression and purification of the PDZ2 domain of the ZO1 protein involved subcloning its encoding construct into a pET28b + plasmid vector, which was subsequently transformed into *Escherichia coli* BL21 (DE3) cells. Bacterial cells were cultured in LB medium with 30 μg/ml kanamycin at 37 °C until reaching an OD600 of 0.7 − 0.8. Protein expression was then induced with 0.5 mM IPTG. Following induction, cells were allowed to grow overnight at 25 °C and were subsequently harvested through centrifugation. The resulting pellet was resuspended in a buffer containing 50 mM TrisHCl, 300 mM NaCl, 10 mM imidazole (pH 8.0), along with one antiprotease tablet (Complete EDTA-free, Roche). The suspension was sonicated and centrifuged, and the soluble fraction from the bacterial lysate was applied to a Ni-charged HisTrap Chelating HP (GE Healthcare) column. The equilibration buffer used was 50 mM TrisHCl, 300 mM NaCl, 10 mM imidazole (pH 8.0). Elution was carried out using an ÄKTA-prime system with a gradient of imidazole ranging from 0 to 1 M. Fractions containing the protein were identified through SDS-PAGE. Buffer exchange to 10 mM Hepes, 150 mM NaCl (pH 7.4) was performed using a HiTrap Desalting column (GE Healthcare). The protein’s purity was assessed via SDS-PAGE. Site-directed mutagenesis was accomplished using the QuikChange mutagenesis kit (Stratagene) following the manufacturer’s instructions.

### Functionalization of PLGA-PEG-Bis-sulfone NPs with wt PDZ2-ZO1

After the incubation, a solution of PDZ2-ZO1 was added in order to reach a final concentration of 200 μM of equivalent of this domain. The final product was incubated at T_amb_ overnight at pH 6 and finally characterized via HPLC. The whole procedure and final construct obtained, named PDZ2-f-NPs, was finally patented (provisional national patent application, No 102023000023964, https://www.sib.it/, October 2023)).

### Synthesis of 6-Coumarin-loaded NPs

6-Coumarin-loaded NPs were made dissolving 5 mg of 6-Coumarin (Sigma-Aldrich, Saint Louis, MO, USA) in 300 μl of a solution of 30 mg of PLGA-PEG-Bis-sulfone in THF. This solution was added dropwise to 7 ml of stirring water. The reaction was left to proceed for 3 h and then they were collected and centrifugated to be subsequently used for further analysis.

### High performance liquid chromatography (HPLC)

To understand the purity of PDZ2-f-NPs, High Performance Liquid Chromatography (HPLC) was performed. All the measurements were made using a Thermo Finnigan Surveyor HPLC using an Agilent ZORBAX RRHD SB300-C8, 12.5 × 2.1 mm, 5 μm as column.

The mobile phases used consisted of 0.1% Trifluoroacetic acid (TFA) (Sigma-Aldrich, Saint Louis, MO, USA) in H_2_O for mobile phase A and 0.1% TFA in 80% Acetonitrile (ACN) (Merck, Darmstadt, DE) for mobile phase B. A gradient elution was conducted at a flow rate of 350 μl/min, with 100 μl of the sample injected using an autosampler set at 10 °C. The column temperature was optimized to 70 °C. Detection of the eluent’s absorbance was performed at both 214 nm and 280 nm.

### Surface activation, ligand immobilization and binding (PDZ2-f-NPs vs SARS-CoV-2 E-protein)

The interaction between the C-terminal portion of the Envelope protein (ligand) and purified PDZ2-f-NPs (analyte) was assessed using the Surface Plasmon Resonance (SPR) technique, employing a Biacore X100 instrument (Biacore, Uppsala, Sweden). The N-terminal biotinylated peptides (ligand, VKNLNSSRVPDLLV^12^) were sourced from GenScript (Piscataway, NJ, USA), and immobilized on a Sensor Chip SA pre-coated with streptavidin from Biacore AB. The ligand immobilization procedure adhered to the manufacturer’s instructions, targeting 1000 response units for ligand immobilization. The running buffer employed was Hepes-buffered saline-EP 1× (HBS-EP 1X), containing 10 mM Hepes (pH 7.4), 0.15 M NaCl, 3 mM EDTA, and 0.05% v/v Surfactant P20 from Biacore AB.

Analytes were dissolved in the running buffer, and binding experiments were conducted at 25 °C with a flow rate of 30 μl/min. The association phase between the ligand and analyte was monitored for 180 s, followed by a dissociation phase lasting 300 s. The highest concentration used for PDZ2-f-NPs was 30 μM (in equivalents of proteins). Concentrations in the SPR assay were achieved through successive dilutions: 30 μM, 15 μM, 7.5 μM, 3.75 μM, 1.875 μM, 1 μM, 0.5 μM, 0.25 μM, and 0.0625 μM. To regenerate the chip’s surface, complete dissociation of the active complex was achieved by introducing 2M NaCl for 30 s before initiating each new cycle. When the experimental data met the quality criteria, data analysis was conducted using the Biacore X100 Evaluation Software. An affinity steady-state model was employed to fit the data since kinetic parameters fell outside the instrument’s measurement range, but an equilibrium signal of interaction was distinctly observed. As a result, the specific K_D_ (dissociation constant) was determined, along with a confidence interval associated with a standard error value to mitigate potential biases.

### Dynamic light scattering (DLS)

The effective average size and the polydispersity index of the development NPs were evaluated using the Zetasizer Nano S (Malvern Instruments, Malvern, UK). NPs were diluted with distilled water and the measurements were carried out using the Dynamic Light Scattering (DLS) mode at 25 °C. The obtained results are the average of at least two analyses on the same sample.

### Nanoparticle tracking analysis (NTA)

NTA (nanoparticle tracking analysis) measurements were conducted using a NanoSight LM10-HS system manufactured by NanoSight (Amesbury, United Kingdom). NPs were diluted at a 1:100 ratio in PBS-1X. Subsequently, the sample was introduced into the sample chamber using sterile syringes (BD Discardit II, New Jersey, USA) until the liquid reached the nozzle’s tip. Five recordings, each lasting 60 s, were performed at room temperature. The NTA software was employed to generate high-resolution particle size distribution profiles and determine particle concentration. Dilution factors were utilized to calculate the particle concentration accurately.

### Cell culture

African green monkey kidney (VERO) epithelial cells (ATCC CCL-81) were cultured in Dulbecco’s Modified Eagle’s Medium (DMEM) supplemented with 10% inactivated fetal calf serum (FCS) (Euro-Clone, Milan, Italy), 1% glutamine (EuroClone, Milan, Italy), 1% streptomycin–penicillin antibiotics (EuroClone, Milan, Italy) and incubated in a humidified atmosphere (5% CO_2_ at 37 °C)^48^.

### Measurement of SARS-CoV-2 infection inhibition by PDZ2-f-NPs

African green monkey kidney (VERO) epithelial cells (ATCC CCL-81) were cultured as reported elsewhere and before^48^. Cells were washed with sterile warm Phosphate buffer (PBS), trypsinized and counted. The monolayer was obtained seeding cells in 48 well plates (Nest) at a final concentration of 7 × 10^4^ cell/ml. When a confluent monolayer > 90% was reached, cells were treated with serial dilutions of PDZ2-f-NPs (PDZ2 concentration in the range from 200 to 1.5 µM) in culture medium. Twenty-four hours later, cells were washed with sterile warm PBS and then infected with 0.1 ml of solution containing SARS-CoV-2 (1 × 10^5^ PFU/ml). Two hours later, infection solution was removed, and new fresh DMEM medium (supplemented with 2% FCS, 1 mM glutamine, 1% streptomycin-penicillin antibiotics) was added. Cells were incubated as previously described and infection status was monitored daily^49^. All the experiments that involved SARS-CoV-2 manipulation were carried out in Biosafety level 3 laboratory (BSL3) in the Institute of Microbiology of IRCCS—Fondazione Policlinico Gemelli.

Crystal violet staining was performed to evaluate cell viability and cellular disruption following the SARS-CoV-2 infection and its replication. Briefly, cells were fixed by using 4% paraformaldehyde for 30 min and then stained by using Crystal violet for 30 min. After incubation five washes were carried out and images were acquired by using Cytation instrument^48,49^.

Images were analyzed using the freely available ImageJ version 1.47v (NIH, USA). Every set of tiff images corresponding to crystal violet staining were analyzed through the “Process > Batch > Macro tool”. Each image was converted to 8-bit image. Minimum and maximum thresholds were manually set for each batch of images, to correctly convert areas to white and black, respectively. Prior to perform the “Measure” tool of ImageJ, all the images were processed with the “Smooth” and “Convert to Mask”. The fraction of the area covered by cells is then automatically stored in the results file^48^.

### Confocal microscopy

VERO cells were plated on µ*-*Slide 8 wells to reach a concentration of 6.000 cells/well and treated for 24 h with 6-coumarin-loaded-PDZ2-f-NPs. Subsequently the cells were washed three times with PBS-1× to be later fixed with 5% formalin for 20 min at room temperature. The cells were then permeabilized with methanol for 4 min at – 20 °C and then they were washed 2 times with PBS-1×. Afterward, cells were blocked with a solution of PBS with 5% Bovine serum albumin (BSA) and 0.1% Tween 20 for 1 h. For the staining, at first, we added a solution of 1:200 of primary antibody (β-Actin (*D6A8*) Rabbit mAb) and incubated it for 2 h. The cells were washed with blocked solution and the secondary antibody was added (Cy^TM^3 AffiniPure Goat Anti-Rabbit IgG (H + L), 1:200, 1h). Finally, VERO cells were washed with blocking solution to be later analyzed with both confocal and fluorescence microscopy. Confocal microscopy measurements were carried out using an inverted microscope (Nikon A1 MP+, Nikon, Tokyo, Japan) equipped with a 20 × objective.

## Results and discussion

### High performance liquid chromatography: results and discussion

**Figure 3:**
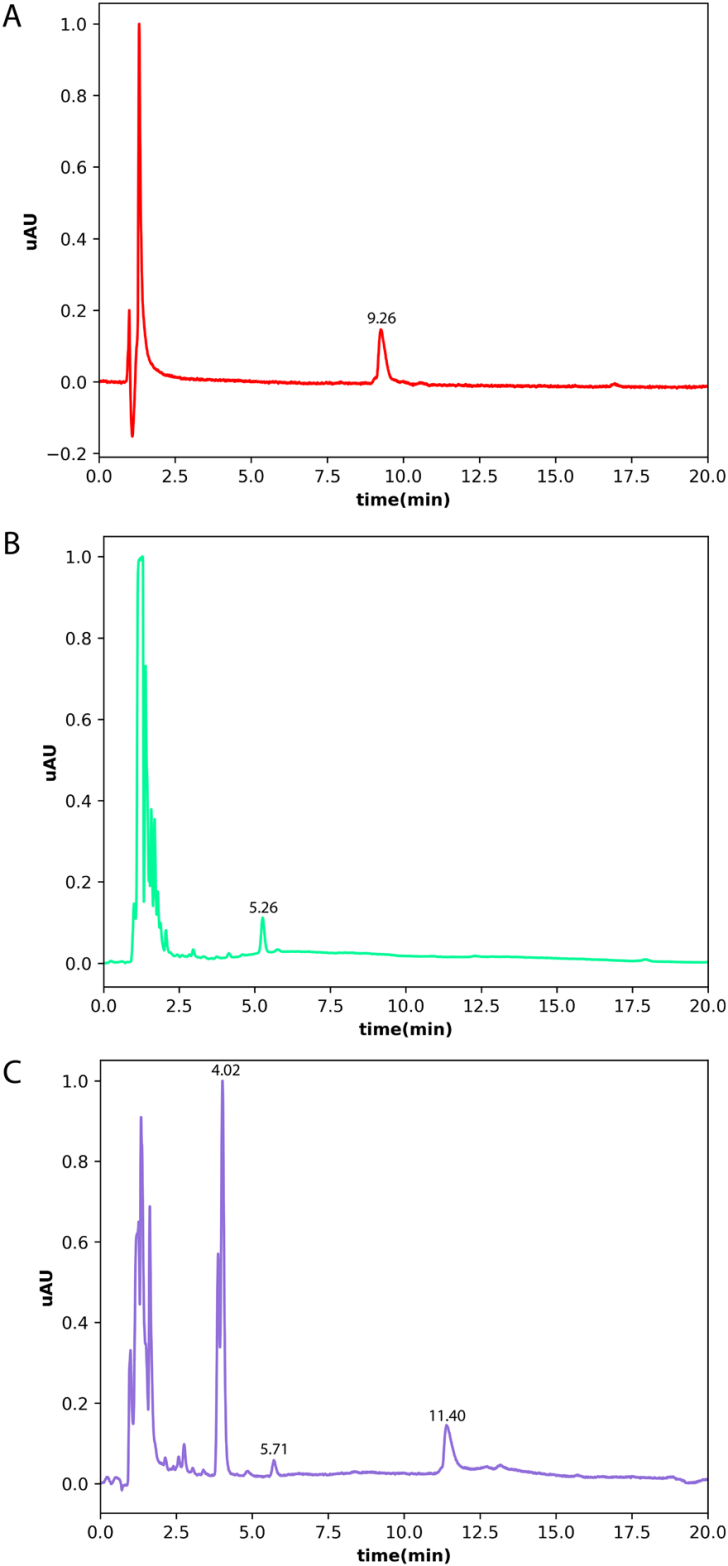
Chromatograms of the different samples. (**A**) Represents the chromatogram of PDZ2-ZO1 (15 μM). (**B**) Shows the spectra of NPs, while (**C**) shows the spectra of PDZ2-f-NPs (15 μM equivalents of PDZ-2).

In Fig. 3, Panel A the chromatogram of a solution of free PDZ2 domain shows a well-defined peak at 280 nm, with an elution time of 9.26 min.

Conversely, standalone polymeric NPs exhibit a distinct 5.26-min elution peak as shown in Panel B of the same figure. Notably, when PDZ2-ZO1 is coupled with NPs, as depicted in Panel C, the signal due to the protein domain extends its elution time to approximately 11.40 min and no detectable signal is present at lower elution times. On the contrary, in the same Panel C, a 5.71-min peak with lower amplitude still remains and probably delineates unfunctionalized NPs, distinct from those bound to the ZO1 domain. Finally, still in panel C, a large peak at 4.02-min is present and corresponds to the formation of p-Toluenesulfonic acid, a byproduct of the reaction. This dataset, due to the non-detectability of a clear signal attributed to the free PDZ2-ZO1 in panel C, suggests that the complexation reaction is complete with a yield that is approximately 100%.

### Dynamic light scattering (DLS) and nanoparticle tracking analysis (NTA): results and discussion

**Figure 4:**
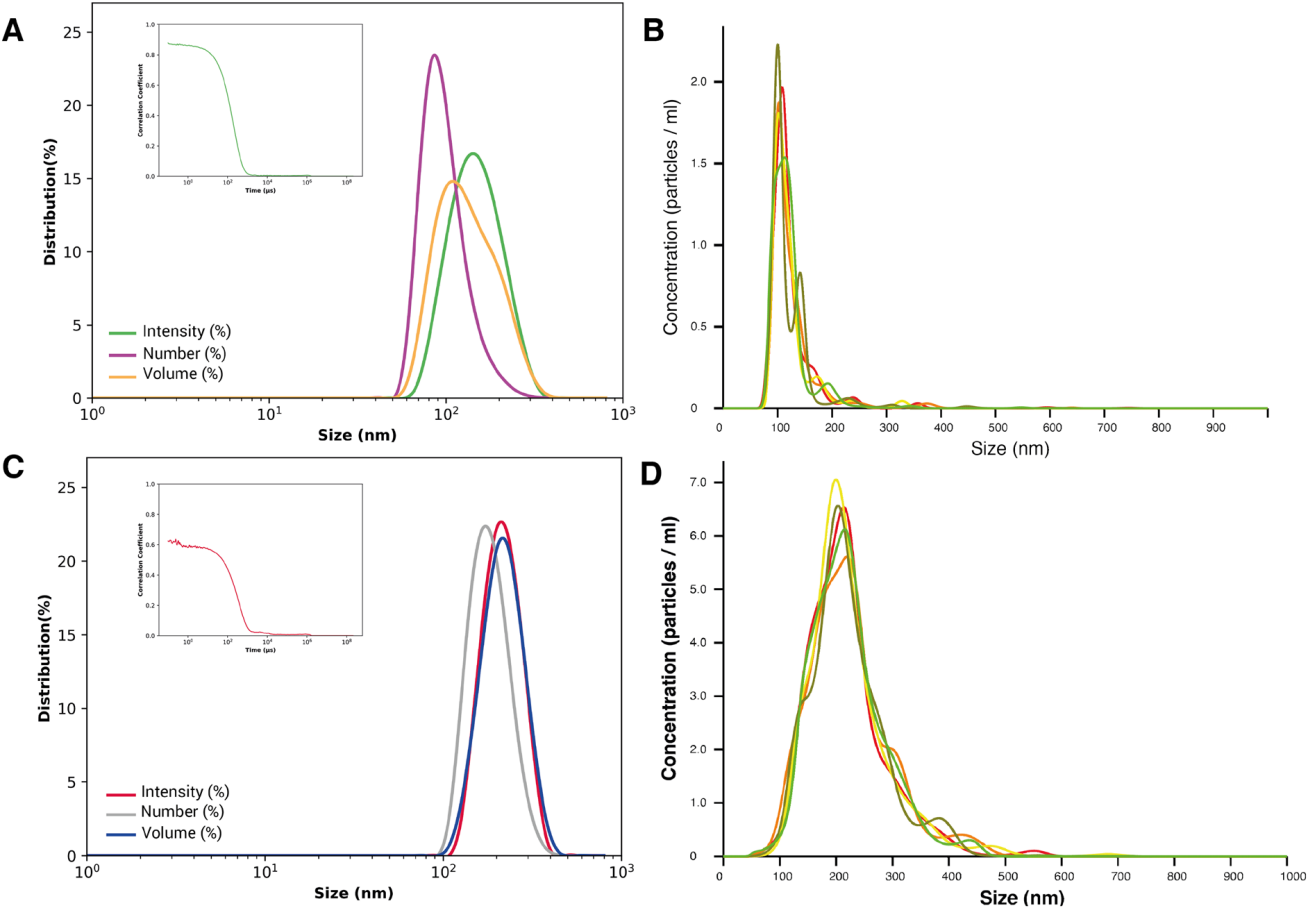
(**A**) Particle size distribution for Intensity (peak = 146 nm), Number (peak = 90 nm) and Volume (peak = 108 nm) and Correlogram of PPB-NPs. (**B**) NTA-size-distribution results for PPB-NPs. (**C**) Particle size distribution for Intensity (peak = 198 nm), Number (peak = 170 nm) and Volume (peak = 230 nm) and Correlogram of of PPB-NPs conjugated with PDZ2-ZO1. (**D**) Size distribution of PDZ2-f-NPs via NTA.

The particle size and size distribution evaluated by the Zetasizer instrument were found to be 140 nm for PPB-NPs and 235 nm for the functionalized nanoparticles: PDZ2-f-NPs (see Fig. 4A,B). The polydispersity index (PDI) obtained for both the preparations were respectively PI = 0.126 for PPB-NPs and PI = 0.22 for the PDZ2-f-NPs. These results confirm that the functionalization process was successful, as demonstrated by the larger size of PDZ2-f-NPs with respect to the free PPB-NPs. Moreover, the higher PDI found for the functionalized ones is also compatible with a larger variability due to the contemporarily presence of functionalized and unfunctionalized particles.

NTA analyses confirmed this trend (see Fig. 4C,D), showing a particles size distribution in the 100 to 300nm range, with a mean value of 122.9 ± 0.8 nm for PPB-NPs and 220.9 ± 0.7 nm for PDZ2-f-NPs. The particle concentration estimated by the software was 1 × 10^10^ particles/ml for both prepaprations.

### SPR analysis to assess the affinity between PDZ2-f-NPs and SARS-CoV-2 E-protein: results and discussion

**Figure 5:**
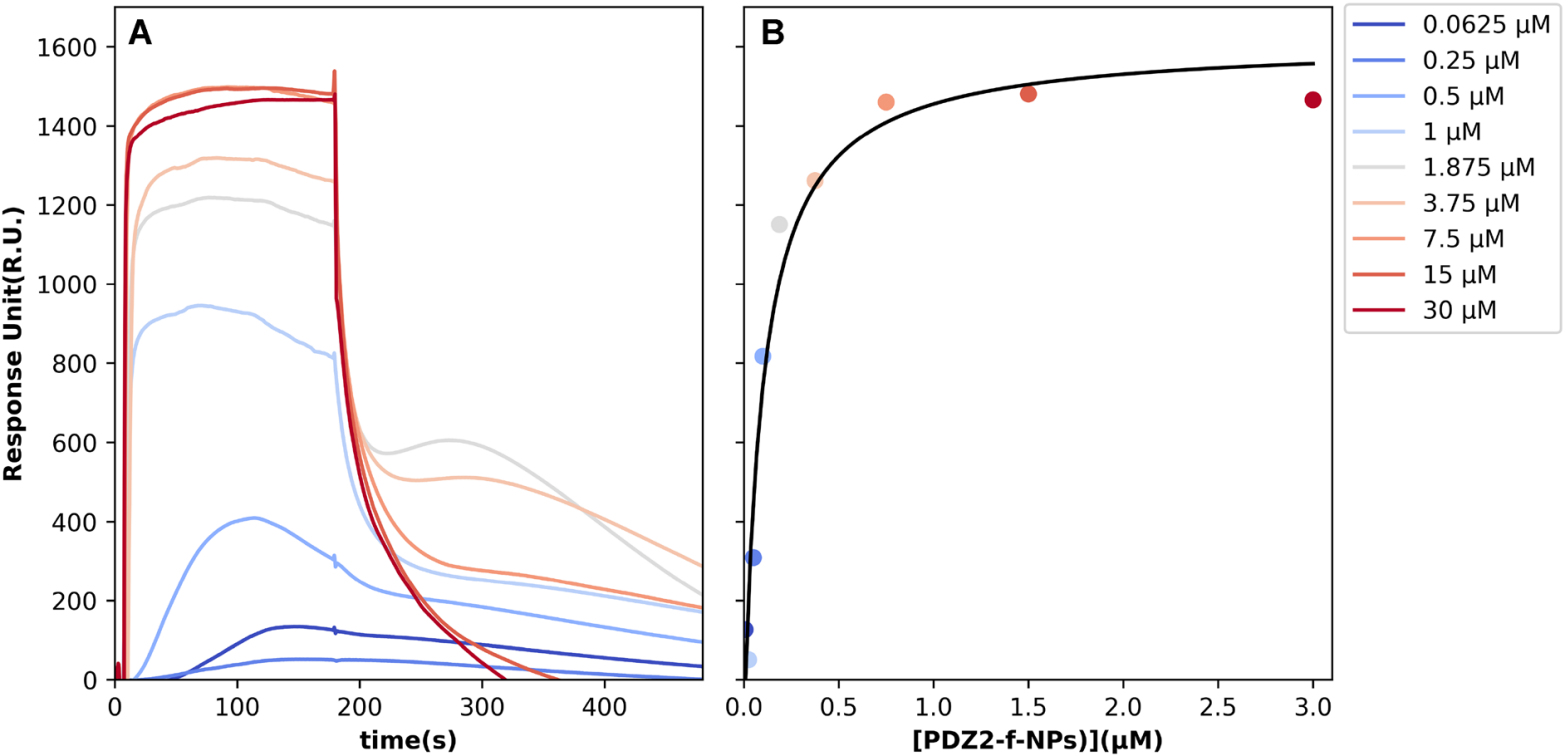
SPR sensorgram and scatchard plot of the interaction between SARS-CoV-2 E protein and the analyte, NPs-PDZ2-ZO1. Biotinylated C-terminal portion of the envelope protein from SARS-CoV-2 was immobilized on SA (streptavidin) sensor chip. (**A**) Experimental curves represent different concentrations of PDZ2-f-NPs used as analyte. The resultant curves were fitted following a single exponential binding model with 1:1 stoichiometry. (**B**) Scatchard plot calculated using Biacore X100 Evaluation Software. The binding isotherm of E-tetradecapeptide and NPs was used to determine the K_D_ between them.

Here, we sought to determine the binding affinity between SARS-CoV-2 E-protein and PDZ2-f-NPs using the Surface Plasmon Resonance technique. SARS-CoV-2 E-protein domain was immobilized on a SA sensor chip and used as ligand in our SPR assay, while, as analyte, we used the PDZ2-f-NPs. The optimal experimental setup was settled, and the analytes were injected at different formal concentrations of the protein domain (30 μM, 15 μM, 7.5 μM, 3.75 μM, 1.8 μM, 1 μM, 0.5 μM, 0.25 μM, 0.0625 μM). As it can be seen in Fig. 5, functionalized NPs show a peculiar behavior either during the association phase either in the dissociation regime. We hypothesized that this trend is due to an avidity effect induced by the spatial co-localization of protein moieties in the functionalized NPs^50^, that, during the dissociation phase, is even more evident, generating a roll-over effect that gives rise to multiple sequential binding event. Indeed, at higher PDZ2-f-NPs concentration, the curve followed the expected pattern, likely indicating the saturation of the active cell with PDZ2 domains, each interacting with its counterpart. Conversely, at lower concentrations of, a distinct trend was observed, marked by noticeable points of change on curve (POCs). We believe that these POCs are the result of the NPs rolling on the SA sensor chip. When the PDZ-2 domain locates and interacts with the ligand, there is a clear increase in Response Units, followed by a subsequent decrease as the NPs detach from the chip surface. We finally analyze this dataset using a Scatchard plot, obtained considering only the maximum signal amplitude of the different sensorgrams, not considering these roll-over effects. Accordingly, we found that PDZ2-f-NPs bind SARS-CoV-2 E-protein with a K_D_ lower (0.97 ± 0.31 μM) than the one measured for the PDZ-2 domain alone 2.1 ± 1.1 μM in previous work.

The data achieved suggest that the interactions between NPs and SARS-CoV-2 E-protein is characterized by a significantly higher affinity compared to the one previously measured^13^. These results are strongly encouraging proving that these nanocarries may be used as a highly promising strategy to overcome the pathogeneses mechanisms induced by SARS-CoV-2 infection.

### Confocal microscopy: results and discussion

**Figure 6:**
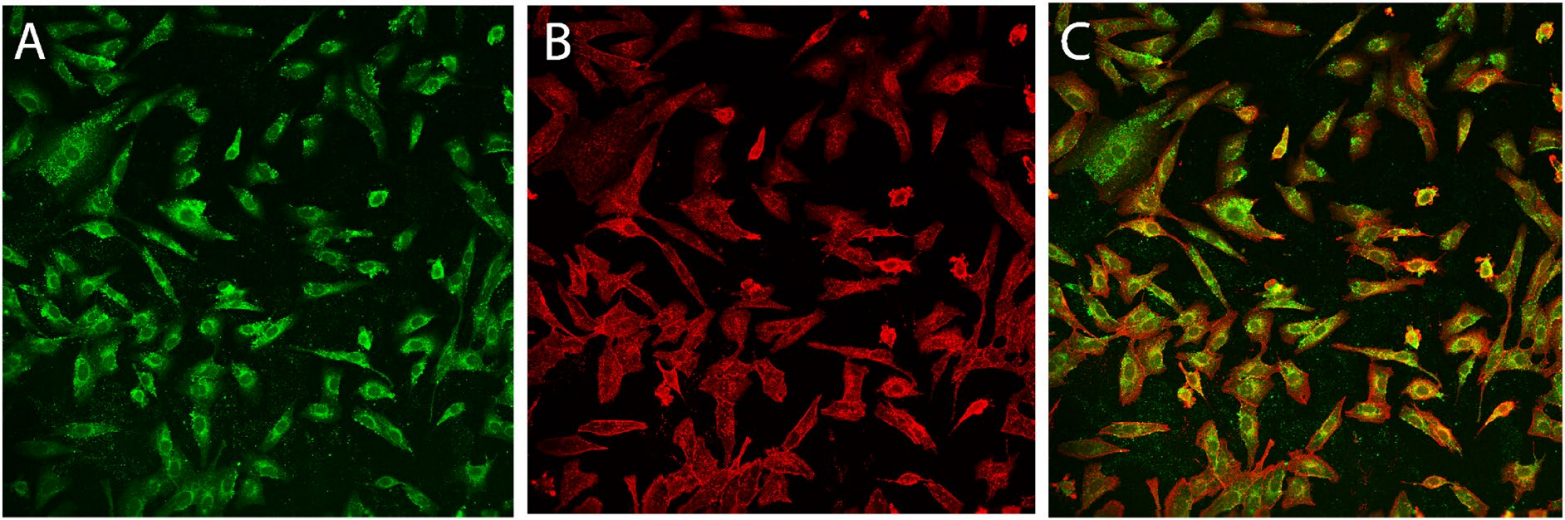
Visualization of 6-Coumarin-loaded-PDZ2-f-NPs (green) in VERO cells via Confocal Microscopy. The different colors represent: β-Actin in red and 6-coumarin-loaded-PDZ2-f-NPs in green.

Confocal Microscopy clearly shows that after 24 h of incubation the internalization of PDZ2-f-NPs occurs (see Fig. 6). The fluorescence signal is predominantly observed in the cytoplasm, probably due to the release of 6-coumarin from NPs. However, confocal microscopy images also reveal a distinct subcellular localization pattern for 6-coumarin-loaded-PDZ2-f-NPs. Indeed, it can be observed that they exhibit a punctate pattern around plasma membrane where they seem to accumulate massively.

The observed localization and distribution pattern of PDZ2-f-NPs provides further insights about the subcellular localization of PDZ2 binding partner.

### Crystal violet staining and infection with SARS-CoV-2: results and discussion

**Figure 7:**
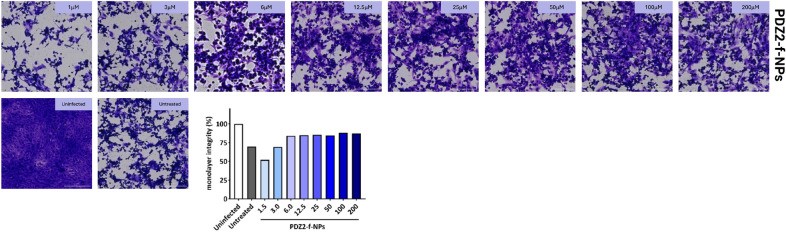
Results of crystal violet staining post-infection of VERO cells with SARS-CoV-2 following treatment with PDZ2-f-NPs.

Nanoparticles biocompatibility was first assessed measuring monolayer integrity following treatment with NPs showing no toxic effect (data not shown). The potential anti-viral activity of the PDZ2-f-NPs against SARS-CoV-2 was then evaluated in VERO cells pre-treated with serial dilution of NPs. Not treated and not infected cells were used as controls. Viral infectivity was measured 48 h after infection, when cells were fixed and stained by using crystal violet (see Fig. 7). PDZ2-f-NPs cell conditioning (concentration ranged from 200 to 6 µM) showed a significant protective effect against SARS-CoV-2. Indeed, monolayer integrity resulted comparable among samples and ~ 85% in comparison with not infected cells. Conversely, any ability to reduce the virus mediated cytotoxicity was observed when VERO cells were pre-treated with low concentration (3 µM and 1.5 µM) of the NPs. This result suggested a promising activity of PDZ2-f-NPs to reduce SARS-CoV-2 mediated cellular damage blocking/interfering with viral intracellular processed triggered by E protein.

## Conclusions

The COVID-19 pandemic has underscored the urgent need for novel antiviral strategies against SARS-CoV-2. While attention has primarily focused on the Spike protein, our research highlights the pivotal role of the SARS-CoV-2 E-protein^4,51–56^, particularly its C-terminal portion containing a PDZ-binding motif (PBM)^5,12,13^. This PBM interacts with human tight junction protein ZO-1’s PDZ2 domain^13,26^, forming the basis for our innovative adjuvant therapeutic strategy. We’ve developed PLGA-PEG-Bis-sulfone nanoparticles (PPB-NPs) externally functionalized with ZO1’s PDZ2 domain, validated through comprehensive analytical techniques including FT-IR (see S1), ^1^H-NMR (see S2), NTA, DLS, and HPLC (see Figs. 3, 4). SPR results reveal the remarkable impact of our approach (see Fig. 5). Through the conjugation of the PDZ2 domain to these NPs, we have effectively augmented the binding affinity between the viral E protein and PDZ2 (see Fig. 5)*.* Our in vitro investigations studies on VERO cells show efficient NPs internalization without cytotoxic effects, establishing a promising foundation for their utilization as drug delivery vehicles (see Fig. 6). Additionally, these NPs exhibit potential in counteracting virus-induced virulence. These findings hold great promise for the development of targeted adjuvant antiviral therapies that not only hinder virus entry and replication but also effectively attenuate the associated virulence factors, potentially revolutionizing our ability to combat viral infections. Indeed, we highlight an alternative intervention against SARS-CoV-2 infection based on a high conserved structural protein, and on the other side we provide an innovative and modulable approach to be applied against other viral pathogens^48,49^. This biotechnological platform is also available for transferring antiviral compounds in the internal polymeric core, thus exerting a synergic proper antiviral effect combined with this adjuvant strategy. Further studies are needed to properly investigate this approach.

### Supplementary Information

Supplementary Information.

## Data Availability

The datasets during and/or analysed during the current study available from the corresponding author on reasonable request. The data that support the findings of this study are available within the article. The raw data are available from the corresponding author upon reasonable request.
