# Supplementary material for: PDZ2-conjugated-PLGA nanoparticles are tiny heroes in the battle against SARS-CoV-2
Source: Sci Rep. 2024 Jun 6;14:13059. doi: 10.1038/s41598-024-63239-w (PMC11156922; doi:10.1038/s41598-024-63239-w)
Supplement: Supplementary file 1 — Supplementary Information. [file 41598_2024_63239_MOESM1_ESM.docx]

Noah Giacon^1$^, Ettore Lo Cascio^1$^, Valeria Pennacchietti^2^, Flavio De Maio^1,3^, Giulia Santarelli^3^, Diego Sibilia^4^, Federica Tiberio^4^, Maurizio Sanguinetti^1,3^, Wanda Lattanzi^4,5^, Angelo Toto^2*^ and Alessandro Arcovito^1,5*^

**“PDZ2-conjugated-PLGA Nanoparticles are Tiny Heroes in the Battle Against SARS-CoV-2”**

^1^Dipartimento di Scienze Biotecnologiche di Base, Cliniche Intensivologiche e Perioperatorie, Università Cattolica del Sacro Cuore, Largo F. Vito 1, 00168 Roma, Italy.

^2^Dipartimento di Scienze Biochimiche "A. Rossi Fanelli", Sapienza Università di Roma, P.le A. Moro 5, 00185, Rome, Italy - Laboratory affiliated to Istituto Pasteur Italia - Fondazione Cenci Bolognetti, Italy.

^3^Dipartimento di Scienze di Laboratorio e Infettivologiche, Fondazione Policlinico Universitario "A. Gemelli", IRCCS, Largo A. Gemelli 8, 00168 Roma, Italy.

^4^Dipartimento di Scienze della Vita e Sanità Pubblica, Università Cattolica del Sacro Cuore, Largo F. Vito 1, 00168 Rome, Italy.

^5^Fondazione Policlinico Universitario "A. Gemelli", IRCCS, Largo A. Gemelli 8, 00168 Roma, Italy.

^$^ these authors contributed equally to this work and share first authorship

* Corresponding authors: Alessandro Arcovito, alessandro.arcovito@unicatt.it, Angelo Toto, angelo.toto@uniroma1.it

**SUPPLEMENTARY MATERIALS**

**Fourier-transform infrared spectroscopy (FT-IR) – Materials and Methods**

Fourier-transform infrared spectroscopy (FT-IR) was employed to characterize PLGA-OH, PLGA-PEG-NH2, and PLGA-PEG-Bis-Sulfone. The FT-IR spectra were obtained at room temperature utilizing a Spectrum One FTIR spectrophotometer manufactured by Perkin Elmer (Norwalk, CT, USA). An Attenuated Total Reflection (ATR) holder was utilized for data acquisition. The spectral data was collected over the wavelength range of 4000-550 cm^-1^, with a resolution of 4 cm^-1^.

To ensure data accuracy, the ATR crystal was rigorously cleaned with either ethanol or DCM before each measurement session. A background spectrum was systematically recorded using an empty "cell" and was subsequently subtracted from the spectrum of each analyzed sample to eliminate any interfering signals.

The spectra were initially recorded in transmission mode using the "Spectrum v.5.0.1 PerkinElmer software" and were later converted into ASCII format for subsequent analysis using Python.

**Nuclear Magnetic Resonance (NMR) – Materials and Methods**

To investigate the PLGA-PEG-Bis-Sulfone polymer, Hydrogen Nuclear Magnetic Resonance (^1^H-NMR) spectroscopy was employed. The ^1^H -NMR spectrum was acquired using a JEOL ECZ operating at 600 MHz instrument in a deuterated DMSO solution. Chemical shifts (δ) were reported in parts per million (ppm), with the peak at 2.54 ppm of DMSO serving as the internal standard.

**Fourier-transform infrared spectroscopy – Results and Discussion**

The FT-IR spectra for all the samples are presented in figure S1. In Panel A (Fig. S1), the PLGA spectrum is depicted and serves as a reference. It illustrates the stretching vibrations of both carbonyl groups (C = O, at 1748 cm^-1^) and the stretching vibrations of ester groups (C-O and C-O-C, at 1169 and 1087 cm^-1^, respectively). Moving on to Panel B, we have the PLGA-PEG-NH2 spectrum. In this spectrum, the vibrational band of C-O-C in PEG ethers coincides with the C-O-C stretching of PLGA esters. Additionally, the characteristic stretching vibrations of PEG C-H bonds can be observed in the wavelength range of 3000-2874 cm-1.

Finally, Panel C in Fig. X displays the PLGA-PEG-Bis-Sulfone spectrum. This spectrum exhibits all the representative peaks observed in the PLGA-PEG-NH2 copolymer described earlier, along with an additional peak at 1719 cm^-1^. This new peak is likely attributed to the ketone group in the Bis-Sulfone compound.


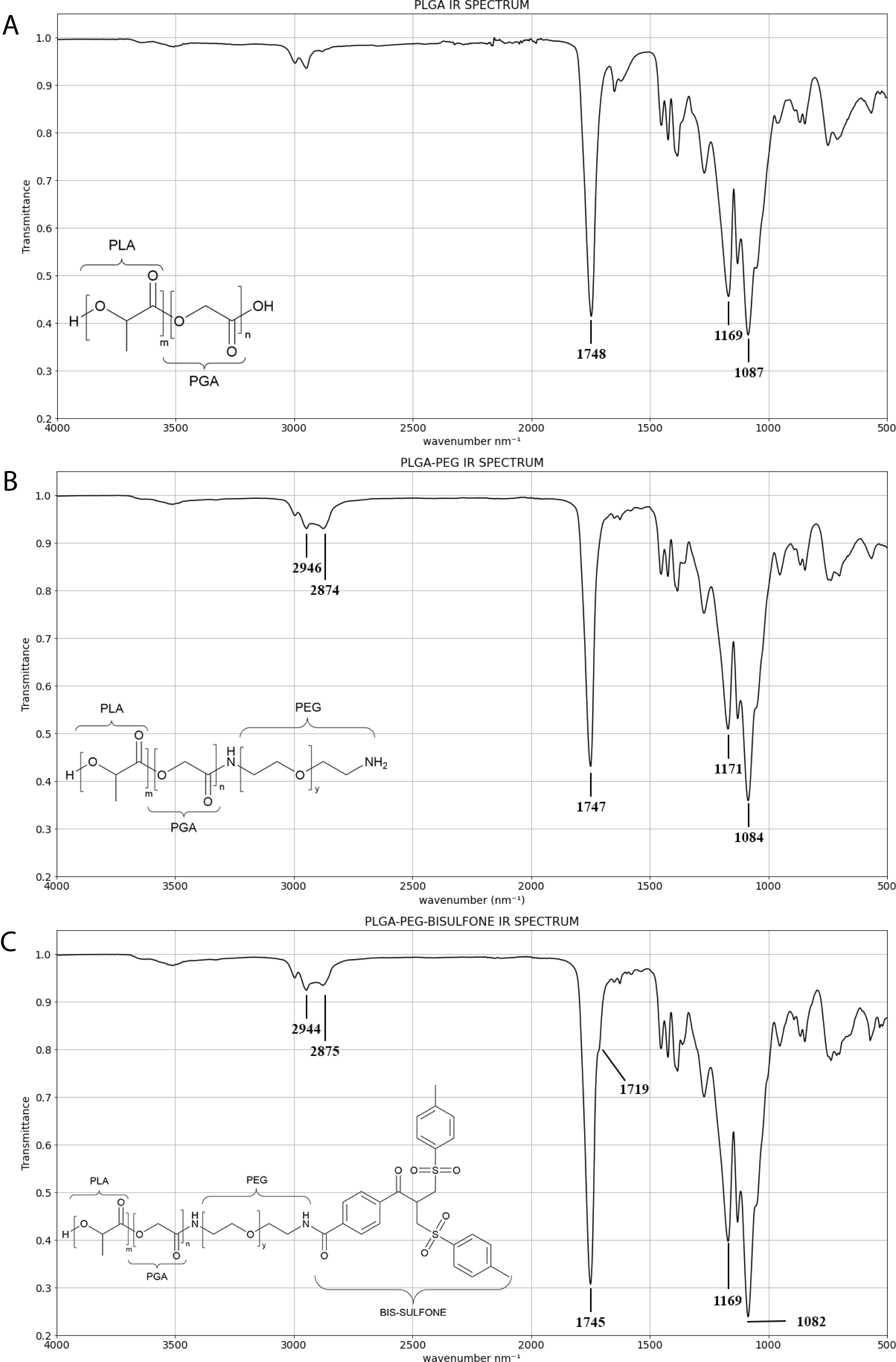


**Figure S1.** **Normalized FT-IR Spectra of the different polymers**. A) PLGA Spectrum, B) PLGA-PEG-NH2 Spectrum, C) PLGA-PEG-Bis-Sulfone Spectrum.

**Nuclear Magnetic Resonance (NMR) – Results and Discussion**

Fig S2. displays ^1^H-NMR spectrum of PLGA-PEG-Bis-sulfone co-polymer. The peaks “a” (1.4 ppm, CH3), “b” (5.2 ppm, CH) and “c” (4.85 ppm, CH2) are associated with PLGA. Peak “d” (3.5 ppm, CH2) is related to PEG, and the peaks in “e” (7 - 8.5 ppm) represent the aromatic groups of Bis-sulfone. ^1^H-NMR spectrum also shows two characteristic peaks respectively at 2.5 ppm and 3.11 ppm due to the presence of DMSO, used as solvent, and methanol impurity.


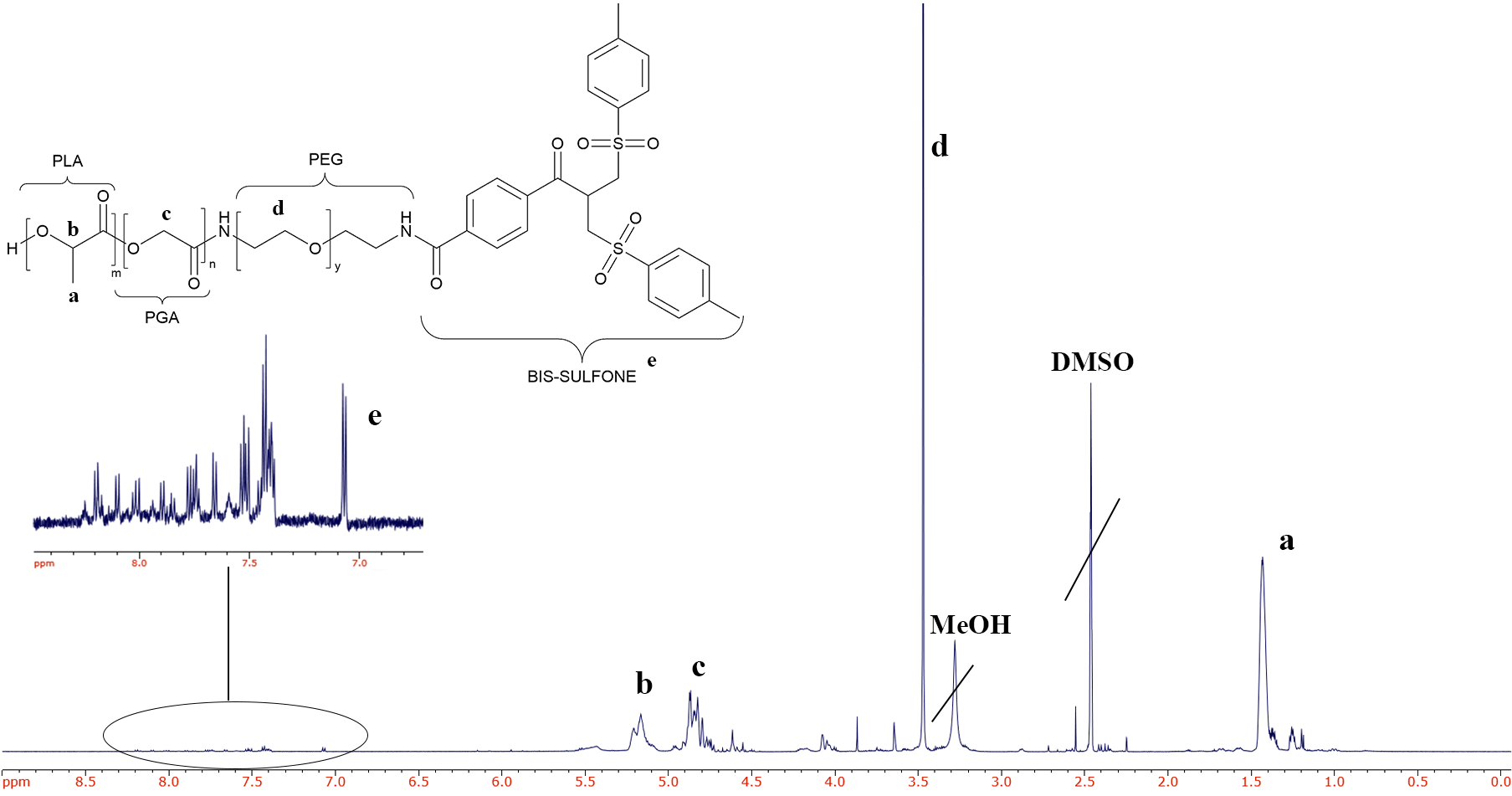


**Figure S2**. ^1^H-NMR Spectrum of the PLGA-PEG-Bis-Sulfone polymer.
